# Persistent cough due to cough variant asthma following upper respiratory tract infection treated by traditional Chinese medicine: A case report

**DOI:** 10.1097/MD.0000000000043957

**Published:** 2025-08-22

**Authors:** Shu-Xuan Li, Zhen Wang

**Affiliations:** a The First School of Clinical Medicine, Zhejiang Chinese Medical University, Hangzhou, Zhejiang Province, China; b The First Affiliated Hospital of Zhejiang Chinese Medical University (Zhejiang Provincial Hospital of Traditional Chinese Medicine), Hangzhou, Zhejiang Province, China.

**Keywords:** case report, cough variant asthma, traditional Chinese medicine

## Abstract

**Rationale:**

Cough variant asthma (CVA) is one of the primary causes of chronic cough, significantly impacting quality of life. Although inhaled corticosteroids–long-acting beta agonists are the first-line treatment, their limitations—such as prolonged treatment duration, high relapse rates after discontinuation, and suboptimal efficacy in some patients—highlight the need to explore alternative treatment options. Traditional Chinese medicine (TCM), with its holistic framework, syndrome differentiation-based treatment, and emphasis on preventing relapse, offers a promising strategy for complementary therapy. This case report aims to evaluate the potential efficacy of personalized TCM protocols for CVA patients who have not responded to conventional treatment.

**Patient concerns:**

A CVA patient with poor medication compliance developed persistent airway inflammation after an upper respiratory tract infection, resulting in worsening of existing cough symptoms and poor response to standard western medical treatment.

**Diagnoses:**

The patient was diagnosed with CVA, and the Chinese medicine diagnosis was cough and wind-heat offending the lung syndrome.

**Interventions:**

The patient received a Chinese medicine decoction. The principle of TCM treatment is to clear heat, dispel wind, relieve the pharynx, and relieve cough.

**Outcomes:**

After 2 weeks of Chinese medicine treatment, the patient’s visual analogue scale, cough evaluation test, Leicester cough questionnaire, and traditional Chinese medicine criteria scores improved. All key indicators reflecting small airway function (forced expiratory flow at 50% of vital capacity, forced expiratory flow at 75% of vital capacity, and maximal mid-expiratory flow) showed sustained and clinically significant improvements. Additionally, the treatment process was safe, with no adverse reactions or abnormal changes in liver, kidney, or hematological parameters observed. Overall, this TCM treatment was safe and effective for this CVA patient.

**Lessons:**

This case demonstrates the potential of TCM as a viable option for managing CVA, particularly in cases suboptimally controlled by conventional therapy. The “one person, one prescription” approach, targeting both pathogenic factors and systemic regulation, may address the multifactorial nature of CVA and contribute to sustained remission. Rigorous larger-scale studies are warranted to validate these findings.

## 1. Introduction

Cough variant asthma (CVA) is a type of atypical asthma in which chronic cough is the only or main clinical manifestation without significant wheezing or shortness of breath; however, airway hyperresponsiveness is present. According to statistics, it accounts for 25% to 42% of chronic cough cases and is the most common cause of chronic cough in China.^[1]^ CVA is mainly characterized by an irritating dry cough, which is predominant at night and in the early hours of the morning, and is usually triggered by exposure to various allergens, viral respiratory infections, cold air, irritating gases, and other factors.^[2]^ The diagnosis of CVA relies on clinical signs and symptoms and objective tests for variable airflow limitation: Positive bronchodilator test, defined as an increase in forced expiratory volume in 1 second (FEV1) of ≥12% and an absolute increase in FEV1 of ≥200 mL after inhalation of a bronchodilator or an increase in FEV1 of ≥12% and an absolute increase in FEV1 of ≥200 mL after 4 weeks of anti-inflammatory treatment with ICS, except for respiratory tract infections within 4 weeks. A positive bronchial provocation test, defined as a ≥20% decrease in FEV1 after inhalation of a provocative agent, suggesting the presence of airway hyperresponsiveness, except for respiratory infections within 4 weeks. Mean daily diurnal variation in peak expiratory flow >10%. A diagnosis of CVA is made if any of the above clinical symptoms are present with evidence of variable airflow limitation, and if other conditions causing the cough are excluded.^[3]^ Currently, the principles of treatment for CVA are the same as those for typical asthma, with inhaled corticosteroids-long-acting beta agonists recommended as the drug of choice and a treatment duration of 8 weeks or more.^[4]^ For patients with severe airway inflammation, treatment with leukotriene receptor antagonists may be added or short-term treatment with oral corticosteroids.^[5]^

Although the current treatment regimens are effective in most patients, they must be used for an extended period. In addition, studies have shown that approximately 30% of patients with CVA respond poorly to standard treatment, with recurrent coughing episodes, and some eventually progress to asthma; moreover, more than half of the patients with CVA have small airway dysfunction that persists despite short-term asthma treatment and has unique clinical and pathophysiological features.^[6]^ The recurrence of cough in well-controlled patients after discontinuation of the drug, decrease in drug sensitivity due to prolonged use, poor patient compliance, and oral fungal infections caused by improper use of the drug suggest that there are still limitations in current Western medical treatment.^[7]^ In recent years, traditional Chinese medicine (TCM) has played a better role in the treatment of CVA, compensating for the shortcomings of Western medicine through the advantages of holistic diagnosis, multi-targeted intervention, regulation of constitution, and prevention of recurrence, etc.^[8]^ Therefore, we would like to share a case report here, which will make the clinical application of TCM more standardized and accurate and provide more choices for the treatment of CVA.

## 2. Case report

### 2.1. Clinical information

The patient, is male, 35 years old. In October 2022, cough variant asthma was diagnosed at the First Affiliated Hospital of Zhejiang Chinese Medical University (Zhejiang Provincial Hospital of Traditional Chinese Medicine). During this period, the patient did not regularly use budesonide–formoterol inhalation. He experienced recurrent coughing after contracting influenza A in December 2024, with poor results from Western cough suppressants, phlegmolytic drugs, and other Chinese medicines, so he visited our outpatient clinic in February 2025. At that time, the patient was coughing day and night, especially at night, which was aggravated by irritating gases, with little white sputum, accompanied by a dry and itchy pharynx. The routine blood test results were normal. Pulmonary function tests: lung ventilation was normal, bronchial provocation test (‐), bronchodilatation test (‐); where FEV1 improved by 8.6% from prediastolic with an absolute increase of 260 mL, and forced expiratory flow at 25% of vital capacity % pred = 89.7%, forced expiratory flow at 50% of vital capacity (FEF50) % pred = 78.5%, forced expiratory flow at 75% of vital capacity (FEF75) % pred = 69.8%, maximal mid-expiratory flow % pred = 74.2%, suggesting decreased small airway function. Specialized examination: T 36.7°C, HR 85 beats/min, R 19 beats/min, BP 125/87 mmHg, normal thorax, no widening of the intercostal space, clear sounds on percussion in both lungs, clear breath sounds, no dry or wet rales, and no rales. Chinese medicine examination revealed a red tongue body with a thin white coating accompanied by a rapid pulse.

The patient had no history of food or drug allergies.

### 2.2. TCM therapy

Chinese medicine diagnosis: Cough; wind-heat offending the lung syndrome.

Treatment principles: Clearing heat, dispelling wind, and relieving pharynx and cough.

The prescription is as follows: Jinyinhua (*Flos Lonicerae*) 15 g, Lianqiao (*Fructus Forsythiae*) 10 g, Huangqin (*Radix Scutellariae*) 12 g, Pugongying (*Herba Taraxaci*) 12 g, Xuchangqing (*Radix Cynanchi Paniculati*) 9 g, Jiangcan (*Bombyx Batryticatus*) 12 g, Wumei (*Fructus Mume*) 9 g, Qianhu (*Radix Peucedani*) 12 g, Jiegeng (*Radix Platycodonis*) 12 g, Kuxingren (*Semen Armeniacae Amarum*) 15 g, Zhebeimu (*Bulbus Fritillariae Thunbergii*) 12 g, Zisuzi (*Fructus Perillae*) 12 g, Xiqingguo (*Fructus Chebulae Immaturus*) 10 g, Shegan (*Rhizoma Belamcandae*) 12 g, Houpo (*Cortex Magnoliae Officinalis*) 12 g, Cangzhu (*Rhizoma Atractylodis*) 12 g, and Zisugeng (*Caulis Perillae*) 12 g.

One dose per day, totaling 300 ml, was taken separately in the morning and evening, half an hour after meals, for 2 weeks.

### 2.3. Treatment outcomes

After 2 weeks of Chinese medicine treatment, the patient’s subjective symptom scores improved significantly (Table 1). The visual analog scale score for cough severity decreased significantly from 6 points (moderate cough) before treatment to 2 points (mild cough) after treatment, clearly indicating a substantial reduction in the severity of cough experienced by the patient. The cough evaluation test score decreased from 19 points before treatment to 11 points, further confirming a notable reduction in cough-related distress. Additionally, the Leicester cough questionnaire score improved from 11.81 points to 14.61 points, indicating a substantial improvement in the patient’s quality of life. The traditional Chinese medicine criteria decreased from 10 points before treatment to 6 points, indicating that the patient’s traditional Chinese medicine syndromes (such as coughing, phlegm production, itchy pharynx and shortness of breath) were effectively alleviated, highlighting the advantages of herbal medicine in regulating overall condition and syndrome differentiation and treatment (Appendix 1, Supplemental Digital Content, https://links.lww.com/MD/P716). Additionally, all key indicators reflecting small airway function (FEF50, FEF75, and maximal mid-expiratory flow) showed consistent and clinically significant improvements, with particularly notable improvements in FEF50 and FEF75 (Table 2). This provides objective physiological evidence for the improvement in clinical symptoms (cough) and quality of life, demonstrating that TCM treatment effectively alleviates the characteristic small airway inflammation and/or spasm in CVA patients.

**Table 1:** Comparison of scale scores before and after treatment.

	VAS	CET	LCQ	TCMC
Before treatment	6	19	11.81	10
After treatment	2	11	14.61	6

CET = cough evaluation test, LCQ = Leicester cough questionnaire, TCMC = traditional Chinese medicine criteria, VAS = visual analogue scale.

**Table 2:** Comparison of pulmonary function before and after treatment.

	FEV1 (% pred)	FEF25 (% pred)	FEF50 (% pred)	FEF75 (% pred)	MMEF75/25 (% pred)
Before treatment	85.2%	89. 7%	78.5%	69.8%	74.2%
After treatment	89.5%	92.4%	83.6%	74.4%	77.1%

FEF25 = forced expiratory flow at 25% of vital capacity, FEF50 = forced expiratory flow at 50% of vital capacity, FEF75 = forced expiratory flow at 75% of vital capacity, FEV1 = forced expiratory volume in 1 second, MMEF75/25 = maximal mid-expiratory flow.

In summary, this TCM treatment demonstrated good efficacy in improving the core symptoms (cough) of CVA patients, enhancing quality of life, and alleviating TCM syndromes. Its efficacy is not only reflected in patients’ subjective experiences but also objectively validated through pulmonary function tests, particularly small airway function indicators. Additionally, the treatment process was safe, with no adverse reactions or abnormal changes in liver, kidney, or hematological indicators observed. Overall, this TCM treatment regimen is effective and safe for this CVA patient, and follow-up indicates that the efficacy is stable.

## 3. Discussion

CVA is the most important cause of chronic cough in China and has a significant impact on people’s daily lives, work, and psychology.^[9]^ TCM treatment has significant efficacy and unique advantages, which can effectively shorten the course of treatment and reduce the recurrence rate through the combination of Chinese and Western medicine.

In Chinese medicine, wind is believed to be the main causative factor of CVA, and wind evil with cold, heat, and dryness invades the lungs. The lung complex is damaged, the function of declaring and purging is out of duty, and cannot drive the evil out of the lungs; for a long time, evil qi in the lungs and lead to the contracture of the airway, and the loss of unimpeded, and there is an increase in the sensitivity and reactivity of the airway, and the symptoms of coughing with the tickle in the throat cannot be stopped when it meets with external triggering factor stimulation.^[10]^ This and the wind of the “good line and several changes” “wind for the long of all diseases” and “wind is itchy” “wind is contracture” characteristics coincide. The general principle of treatment is to clear the wind, promote the lungs, stop the cough, and relieve the pharynx. Treatment methods such as dispersing cold, clearing heat, moistening dryness, expectorating phlegm, and replenishing the deficiency are added according to different symptoms.

The Chinese herbal prescription is from the long-term clinical practice of Prof Linyi Ge, a master of State Medicine, in which Jinyinhua (*Flos Lonicerae*) and Lianqiao (*Fructus Forsythiae*) are pungent, cool, light, and propagandizing, dispersing evils through excretion, clearing heat, and removing toxins as sovereign drugs, which can inhibit inflammatory reactions of the respiratory tract and alleviate coughs induced by viral infections.^[11,12]^ Huangqin (*Radix Scutellariae*) and Pugongying (*Herba Taraxaci*) clear heat and detoxification, eliminating swelling and dissipating knots, enhancing the power of the monarch’s medicine to clear heat, assisting in eliminating the heat of the lungs^[13,14]^; Xuchangqing (*Radix Cynanchi Paniculati*) and Jiangcan (*Bombyx Batryticatus*) to dispel wind and antispasmodic, relieving coughing and asthma, Wumei (*Fructus Mume*) to promote the production of fluid and moisturize the lungs, astringent lung qi, modern pharmacological research has found that the Jiangcan (*Bombyx Batryticatus*) contain protease inhibitors, which can alleviate airway spasm, Xuchangqing (*Radix Cynanchi Paniculati*) and Wumei (*Fructus Mume*) has an antiallergic effect, reducing the hyperreactivity of the airway,^[15–17]^ the above as a minister drug. Qianhu (*Radix Peucedani*) reduces qi and resolves phlegm, evacuates wind-heat; Kuxingren (*Semen Armeniacae Amarum*), Zisuzi (*Fructus Perillae*) reduce the reversal of cough and asthma, Zhebeimu (*Bulbus Fritillariae Thunbergii*) clears heat and resolves phlegm; Jiegeng (*Radix Platycodonis*) promotes lung and phlegm, carries medicines upward, and the above make the functions of dispersing and descending of lung qi are in harmony. Xiqingguo (*Fructus Chebulae Immaturus*) and Shegan (*Rhizoma Belamcandae*) benefit the pharynx and relieve cough, relieve pharyngeal itching and cough, and reduce cough reflex; Houpo (*Cortex Magnoliae Officinalis*) dries dampness and moves qi, calms asthma, and broadens the chest; Cangzhu (*Rhizoma Atractylodis*) dries dampness and strengthens the spleen; Zisugeng (*Caulis Perillae*) regulates qi and neutralizes the middle, and the 3 take care of the spleen and stomach, “eliminating the source of phlegm,” and the above are the adjuvant drugs. Jiegeng (*Radix Platycodonis*) leads the medicine up to the lung meridian, which is also beneficial to the throat and harmonizes all the medicines as an envoy drug. These drugs play a role in clearing heat, dispelling wind, and relieving pharynx and cough.

## 4. Conclusion

Chinese medicine starts with the concept of wholeness, through syndrome differentiation and treatment, and adopts the principle of “one person, one prescription” for treatment, which has been proven to have a good effect on various types of CVA. In this case, a CVA patient whose condition worsened due to a respiratory tract infection experienced significant improvement in symptoms after 2 weeks of TCM treatment, despite previously showing poor response to conventional Western medical treatment.

However, this study has limitations that should be carefully considered: the results are based solely on a single case report. While the findings are encouraging, they are inherently susceptible to random variation and may not be representative of a broader CVA patient population. Additionally, key assessment metrics such as visual analogue scale, cough evaluation test, Leicester cough questionnaire, and traditional Chinese medicine criteria scores rely on patients’ subjective perceptions and are inherently subjective. Although supported by objective lung function tests (e.g., FEF50, FEF75), the primary symptom assessment remains susceptible to reporting bias.

A study with a sample size of 70 is currently underway to comprehensively validate the efficacy of this treatment method, aiming to make a meaningful contribution to the integration of traditional Chinese medicine in CVA management.

## Acknowledgments

Thanks to Prof Linyi Ge for her support in writing the manuscript.

## Author contributions

**Data curation:** Shu-Xuan Li.

**Investigation:** Shu-Xuan Li, Zhen Wang.

**Writing – original draft:** Shu-Xuan Li.

**Writing – review & editing:** Zhen Wang.

## Supplementary Material


