# Supplementary material for: Persistent cough due to cough variant asthma following upper respiratory tract infection treated by traditional Chinese medicine: A case report
Source: Medicine (Baltimore). 2025 Aug 22;104(34):e43957. doi: 10.1097/MD.0000000000043957 (PMC12384995; doi:10.1097/MD.0000000000043957)
Supplement: Supplementary file 1 [file medi-104-e43957-s001.pdf]

Appendix 1 traditional Chinese medicine criteria (TCMC)

level / symptom	none（0 point）	mild（1 point）	moderate（2 points）	severe（3 points）
cough				
phlegm				
itchy pharynx				
shortness of breath				
cough aggravated by heat, cold, or irritating gases				

Note: Each symptom is categorized into 4 grades: none, mild, moderate and severe, corresponding to a score of 0 to 3. The higher the score, the more severe the symptom.
